# The RUBISCO to Photosystem II Ratio Limits the Maximum Photosynthetic Rate in Picocyanobacteria

**DOI:** 10.3390/life5010403

**Published:** 2015-02-04

**Authors:** Jackie K. Zorz, Jessica R. Allanach, Cole D. Murphy, Mitchell S. Roodvoets, Douglas A. Campbell, Amanda M. Cockshutt

**Affiliations:** Department of Chemistry & Biochemistry, Mount Allison University, Sackville, New Brunswick, E4L 1G8, Canada; E-Mails: jkzorz@mta.ca (J.K.Z.); jrallanach@mta.ca (J.R.A.); cdmurphy@mta.ca (C.D.M.); msroodvoets@mta.ca (M.S.R.); dcampbel@mta.ca (D.A.C.)

**Keywords:** *Prochlorococcus*, *Synechococcus*, Photosystem I: Photosystem II: Cytochrome b_6_f, RUBISCO

## Abstract

Marine *Synechococcus* and *Prochlorococcus* are picocyanobacteria predominating in subtropical, oligotrophic marine environments, a niche predicted to expand with climate change. When grown under common low light conditions *Synechococcus* WH 8102 and *Prochlorococcus* MED 4 show similar Cytochrome b*_6_*f and Photosystem I contents normalized to Photosystem II content, while *Prochlorococcus* MIT 9313 has twice the Cytochrome b*_6_*f content and four times the Photosystem I content of the other strains. Interestingly, the *Prochlorococcus* strains contain only one third to one half of the RUBISCO catalytic subunits compared to the marine *Synechococcus* strain. The maximum Photosystem II electron transport rates were similar for the two *Prochlorococcus* strains but higher for the marine *Synechococcus* strain. Photosystem II electron transport capacity is highly correlated to the molar ratio of RUBISCO active sites to Photosystem II but not to the ratio of cytochrome b*_6_*f to Photosystem II, nor to the ratio of Photosystem I: Photosystem II. Thus, the catalytic capacity for the rate-limiting step of carbon fixation, the ultimate electron sink, appears to limit electron transport rates. The high abundance of Cytochrome b_6_f and Photosystem I in MIT 9313, combined with the slower flow of electrons away from Photosystem II and the relatively low level of RUBISCO, are consistent with cyclic electron flow around Photosystem I in this strain.

## 1. Introduction

The picocyanobacteria *Prochlorococcus* and marine *Synechococcus* are the most abundant oxygenic photosynthesizing organisms in the majority of the oligotrophic ocean [1], together contributing 32% to 80% of oceanic primary productivity [2,3,4,5,6]. Their ability to live in regions of low nutrient concentrations gives rise to the prediction that their global distribution will expand in the future with the expected increase in stratification of the world’s oceans due to rising sea temperatures [7,8,9]. Differences in the distributions of marine *Synechococcus* and *Prochlorococcus*, both throughout the globe and throughout the water column, allow these two genera to numerically dominate the majority of the marine environment [10]. Specifically, marine *Synechococcus* live mostly in coastal regions in the upper euphotic zone but can also be found in regions of open ocean, where they are outnumbered by *Prochlorococcus* [1,11]. *Prochlorococcus* resides mainly in the oligotrophic open ocean down to the entirety of the euphotic zone [1]. 

*Prochlorococcus* likely evolved from a shared ancestor within the marine *Synechococcus* radiation about 100–200 million years ago [12]. Members of the *Prochlorococcus* genera have undergone genome streamlining and cell size reduction under the challenges of surviving in a nutrient limited environment [13]. One example of this reduction is that all members of *Prochlorococcus* have lost the genes necessary to produce the nitrogen rich phycobilisome light harvesting complexes found in almost all cyanobacteria [10]. Instead, *Prochlorococcus* uses Prochlorophyte chlorophyll binding (Pcb) proteins that are evolutionarily derived from the low iron inducible Iron Stress Inducible (IsiA) antenna protein of cyanobacteria [10]. Each Pcb protein (~38 kDa) binds 8–11 divinyl chlorophyll a (Chl *a_2_*) and divinyl chlorophyll b (Chl *b_2_*), whereas the αβ-phycobiliprotein dimers (~38 kDa) of *Synechococcus* bind only five tetrapyrrole pigments [10,14], giving rise to different absorption spectra and nitrogen costs for light capture.

These differing adaptations in cell size, genome size and light harvesting strategies between *Prochlorococcus* and marine *Synechococcus* impose differences in their acclimatory and stress responses [15,16]. There are also distinctions within the *Prochlorococcus* genus that result in niche differentiation among ecotypes [1,10,17,18]. Specifically, the *Prochlorococcus* strains that are most deeply branching and, thus, more closely related to marine *Synechococcus*, *Prochlorococcus* MIT 9313 for example, are members of the low or moderate light level ecotypes. These lower light ecotypes reside in the deeper euphotic zone, which receives much less light but relatively higher nutrient concentrations. Conversely, high light level *Prochlorococcus* strains, MED 4 for example, have evolved more recently and reside within the upper levels of the euphotic zone where light levels are higher but nutrient concentrations are extremely low. These more recently evolved, high light, low nutrient strains have the smallest cell and genome sizes within the *Prochlorococcus* radiation. Comparative genomic analysis of these *Prochlorococcus* ecotypes has been reported in depth [2,19,20,21]. The main genomic consequences of *Prochlorococcus* ecotype differentiation manifest in varying pigment ratios, divergent capacities for nutrient uptake between strains, and the acquisition through horizontal gene transfer of genomic islands that confer competitive advantages to particular ecotypes. 

In contrast to genes specific to the *Prochlorococcus* or marine *Synechococcus* genomes, the genes involved in core processes, such as photosynthesis, are highly conserved [20]. The process of photosynthesis in cyanobacteria is mediated by the abundant, large multi-subunit protein complexes photosystem II (PSII), cytochrome b_6_f (cytb_6_f), and Photosystem I (PSI) arranged linearly along a thylakoid membrane [22] After an initial excitatory photon strike electrons are transferred from complex to complex through a series of redox reactions and acceptor/donor intermediates generating a proton gradient in the process. The terminal electron transfer in the photosynthetic electron transport chain is the reduction of NADP+ to NADPH, which is used as a reducing agent in many biosynthetic pathways, notably the carbon fixing Calvin cycle. 

Previous work has included monitoring levels of mRNA transcripts encoding major photosynthetic complexes in cyanobacteria under different experimental treatments [23,24,25]. While measuring mRNA transcript abundance shows factors that cause induction or activation of a particular gene, it does not seamlessly translate to functional protein levels in the cell [25,26,27]. Protein molecules have greater stability than mRNA molecules, which can be differentially degraded, thus introducing a source of potential bias to results [26]. In addition, production of protein is a more energetically expensive process for the cell than production of mRNA. This is of particular importance in organisms growing under mineral nutrient resource limitation. When aiming to determine the present functional state of a cell, or the resources allocated to particular complexes, measuring protein levels gives a better picture of the current cellular status in comparison to other forms of analysis [27,28]. Quantifying absolute protein amounts and standardizing with total protein content can be accomplished by quantitative immunoblotting with global antibodies and internal protein standards [26,29,30]. For our purposes the major photosynthetic protein complexes can be represented by key protein subunits including the PsbA and PsbD proteins of PSII; PetC, the Rieske iron-sulfur protein of Cytb_6_f; and the PsaC protein that forms part of the docking site for ferredoxin or flavodoxin in PSI. Along with these photosynthetic proteins, the Calvin cycle can be represented by RbcL, the large protein subunit, of the enzyme RUBISCO, which catalyzes the rate-limiting step of the Calvin cycle. 

In this study, we quantified the protein composition of the thylakoid membranes in three ecologically relevant species of picocyanobacteria—marine *Synechococcus* WH8102, *Prochlorococcus* MIT 9313, and *Prochlorococcus* MED 4—all grown under similar low light conditions. We seek to elucidate differences in thylakoid function that might be a direct result of niche differentiation in ecotypes.

## 2. Experimental Section 

### 2.1. Culturing

Cultures of *Synechococcus* sp. WH 8102 and *Prochlorococcus* spp. MED 4 and MIT 9313 obtained from the Provasoli-Guillard National Center for Marine Algae and Microbiota were grown at 22–23 °C with innoculation in a 1:4 dilution into PCR S11 and Pro99 media. Cultures were grown under 20–40 µmol photons m^−2^·s^−1^ on a 12:12 light:dark cycle. Culture growth was tracked by daily absorbance measures of 1 mL culture sub-samples (UV-1800 Shimadzu spectrophotometer, Shimadzu, Kyoto, Japan and UVProbe software, version 1.1). Absorbance at 750 nm (A_750_) was subtracted from the absorbance at 680 nm (A_680_) to obtain a scattering-corrected chlorophyll content approximation. These cultures achieved a stable apparent quantum yield of photosynthesis of 0.4 (*Synechococcus*) or 0.6 (*Prochlorococcus*) determined by the ratio of the variable fluorescence (F_V_) to the maximum fluorescence (F_M_) using a Xenon-PAM fluorometer (Walz, Effeltrich, Germany). Exponential growth rates were estimated by measuring the slope of the linear portion of the logarithmic growth curve. Samples for subsequent protein and functional analyses were harvested after 48–72 h of exponential growth.

### 2.2. Whole Cell Protein Extraction and Quantitative Immunoblotting

Once cultures had passed 48–72 h of exponential growth phase with stable (F_V_/F_M_) cell pellets were harvested by centrifugation from 50 mL of each culture and resuspended in 200 µL of protein extraction buffer (1X AEBSF, Roche, Indianapolis, IN, USA and 1X PSB, Agrisera, Vännäs, Sweden). Samples were then homogenized using the CY: 24 × 2 rotor of the FastPrep®-24 Instrument (MPBIO) for two 1 min periods with 1 min of cooling on ice after each treatment. The protein extracts were then centrifuged at 10,000× *g* for 3 min to remove insoluble material and unbroken cells. Protein concentrations of extracts were quantified using the microplate DC protein assay (Bio-Rad, Hercules, CA, USA) with Bovine Gamma Globulin (BGG) as a comparative protein standard. 

The sample preparation, SDS-PAGE, immunoblotting and quantitation protocols have been described in detail previously [30]. Depending on the strain and antibody used, a known amount (1–10 μg) of total cellular protein was loaded in each well. Three to four loads of calibrated standards for each protein (Agrisera) were also loaded on each gel allowing the generation of standard curves that spanned at least one order of magnitude. Primary antibodies (Agrisera) were diluted in 2% ECL advance blocking agent (GE Healthcare) in TBS-T; α-PsbA 1:25,000; α-PsbD 1:25,000; α-PsaC 1:5,000; α-PetC 1:10,000 and α-RbcL 1:10,000. HRP-conjugated goat anti-rabbit (Agrisera) was employed as the secondary antibody at a dilution of 1:40,000. Immunoblots were developed with ECL Advance (GE Healthcare) and images were captured on a VersaDoc Imager (BioRad) and analyzed using Quantity One and Image Lab 3.0 software (BioRad). Comparing the signal in known amounts of each sample with the standard curves (signal plotted *versus* fmoles of standard loaded) allowed determination of fmoles of each protein analyzed per µg total cell protein. See Supplemental Figure 1 for a sample blot, calibration curve and data table.

### 2.3. Physiological Measurements

For analyses of Photosystem II function and down stream electron transport we used Fast Repetition Rate (FRR) Chlorophyll Fluorescence saturation induction and relaxation profiles [31,32,33]. Each sample was analyzed in a Photon Systems Instruments FL 3500 Fluorometer (PSI, Czech Republic) by applying a script to activate FRR inductions after 60 s exposure to a series of incubations under increasing light intensities: 0, 11, 25, 34, 50, 80, and 155 µmol photons m^−2^·s^−1^. For each incubation we applied an FRR induction with the light level active to define F_0_ (after the 0 µmol photons m^−2^·s^−1^ period) or F_S_, the fluorescence in the light acclimated state (after the illuminated periods), and F_M_ (after the 0 µmol photons m^−2^·s^−1^ period) or F_M_', the maximal fluorescence with all PSII closed in the light acclimated state, under the influence of any induction of Non-Photochemical Quenching (NPQ). We fit the induction curve to extract the parameters σ_PSII_ (after the 0 µmol photons m^−2^·s^−1^ period) or σ_PSII_', the effective absorbance cross section serving PSII photochemistry in the light acclimated state and ρ, reflecting excitation connectivity among PSII centres. Immediately following the FRR induction flashlet train we continued probe flashes to track the relaxation of fluorescence back towards F_0_ or F_S_. We fit this curve of PSII re-opening after the saturating flash with a two-phase exponential decay to define τ_1_ and τ_2_, the initial fast and slow decay lifetimes reflecting electron transport away from PSII. We used the reciprocal of τ_2_ as an index for the sustained capacity to remove electrons from PSII. 

After this FRR flash and relaxation applied with background illumination we exposed the samples to a 2 s dark period and the applied a second FRR induction and relaxation to determine F_0_'_(2s)_, the baseline fluorescence with all PSII open but still in a light acclimated state and F_M_'_(2s)_ the maximal fluorescence with all PSII closed in the light acclimated state, along with σ_PSII_'_(2s)_.

We followed [33] to estimate Photosystem II electron transport across the range of light levels as:

e^−^ PSII^−1^ s^−1^ = σ_PSII_'/(F_V_/F_M_) × ΦPSII × I (photons m^−2^·s^−1^)

where σ_PSII_' was measured at the respective light level, F_V_/F_M_ = (F_M_ − F_0_/F_M_), and ΦPSII = (F_M_' − F_S_/ F_M_') measured at the respective light level I to reflect photochemical closure of PSII and induction of any non-photochemical down regulation of PSII under the respective light level. Again following [33] we have recently re-validated this estimator of electron transport rate per PSII centre by near-simultaneously measuring FRR induction curves, steady state Oxygen Evolution and the functional content of Photosystem II using O_2_ flash yields on a set of *Prochlorococcus* and *Synechococcus* culture samples. As expected, we found a strong correlation between our FRR estimate of electron transport per PSII and the near-simultaneous measures based upon O_2_ evolution, with a linear slope of 1.3 and an R^2^ of 0.6 (See Supplemental Figure 2). We fit the PSII light response curves resulting from our FRR measures with a hyperbolic tangent model [34]:

ETR=ETRmax × tanh(I/E_K_)

where E_K_ is the light level at the transition from the light limited to the light saturated regions of the light response curve and I is the instantaneous light level (µmol photons m^−2^·s^−1^).

At the same time as sampling for photophysiology a 1.5 mL sample of culture in exponential phase was mixed with 10 µL of 100X Pluronic F-68 stock to facilitate pelleting and then centrifuged for 5 min at 13,000× *g*. The supernatant was removed and the tube containing the pellet was fully covered in tinfoil. Under dim lighting, the pellet was resuspended in 1 mL of 90% acetone saturated with magnesium carbonate by vortexing. The tube was then placed at −20 °C for 48 h to allow for thorough release of the chlorophyll pigment. Under very dim lighting, the tube was removed from the freezer and centrifuged 5 min at 13,000× *g* to pellet the cell debris and magnesium carbonate. The chlorophyll-containing supernatant was removed and the 390 to 750 nm absorbance spectrum of 0.5 mL of the supernatant was obtained using the Shimadzu spectrophotometer. The baseline was set with 90% acetone without magnesium carbonate. The whole cell suspension absorbance spectrum of each culture was obtained from 390–750 nm using the Olis 14 UV/VIS/NIR spectrophotometer (Olis, Bogart, GA, USA) and Spectral Works/Clarity software, which measured 4 mL of culture in an integrating cavity that corrects for cellular scattering.

## 3. Results and Discussion

### 3.1. Changes in the Photosynthetic Electron Transport Chain Stoichiometry Following Subculturing

**Figure 1:**
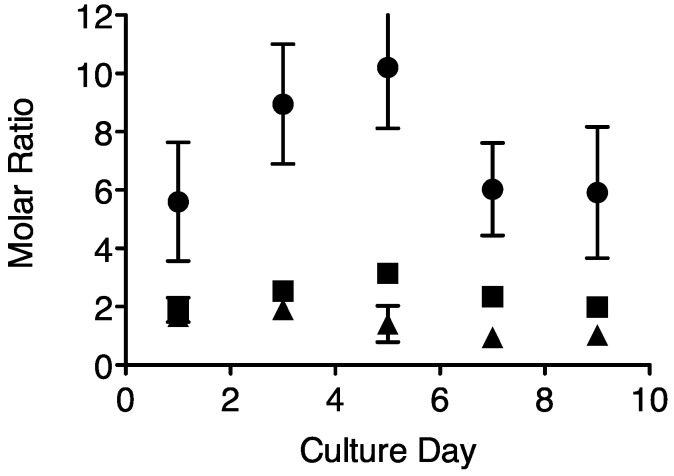
Molar ratios of representative protein subunits PsaC:PsbA (PSI:PSII, circles); PetC:PsbA (Cytb_6_f complex:PSII, squares); and PsbD: PsbA (PSII:PSII, triangles) over time in cultures of *Prochlorococcus* MIT 9313. n = 6 independent determinations, ± SEM.

Figure 1 shows the molar ratios of PSI:PSII, cytochrome b*_6_*f:PSII and RUBISCO:PSII in *Prochlorococcus marinus* MIT 9313 with days in culture following a one in four dilution of the culture into fresh Pro99 medium. The PSI:PSII ratio nearly doubled over the first five days in culture, and then declined. Over this period the quantum yield of PSII (F_V_/F_M_) did not change (data not shown). For consistency, all subsequent measurements were conducted on cells three to four days post dilution; after two to three days of exponential growth.

### 3.2. Molar Quantitations of Photosynthetic Protein Complexes

**Figure 2:**
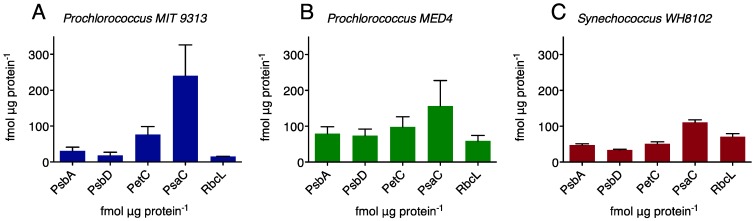
Molar immunoquantitations of representative protein subunits PsbA (PSII); PsbD (PSII); PetC (Cytb_6_f complex); PsaC (PSI) and RbcL (RUBISCO large subunit) (**a**) *Prochlorococcus* MIT 9313; (**b**) *Prochlorococcus* MED 4; (**c**) *Synechococcus* WH8102. n = 6 independent determinations, ±95% confidence interval.

Figure 2 presents the molar contents of representative proteins from PSI, PSII, the cytochrome b*_6_*f complex and RUBISCO in two *Prochlorococcus* strains, MIT 9313 and MED 4 and *Synechococcus* WH 8102 three to four days following subculturing. While these cultures are all monoalgal, they are not axenic, and harbour other bacteria, some of which may promote the growth of the picocyanobacterial cells [15]. For this reason caution must be taken in comparing absolute quantitations of proteins, as the fraction of the total protein isolated that derives from the cyanobacterial cells may vary with the extent of non-cyanobacterial bacteria in culture. As the photosynthetic proteins are only found in the cyanobacterial cells, we are able to confidently report the molar stoichiometries of the protein complexes (see Table 1). The three picocyanobacterial strains have remarkably different photosynthetic apparatus stoichiometries despite being grown under common conditions. When normalized to the PsbA proxy for PSII content, the PSU stoichiometry (PSII:Cytb*_6_*f:PSI:RUBISCO) is 1:2.5:9.0:0.5 for MIT 9313; 1:1.3:2.0:0.8 for MED 4; and 1:1.1:2.3:1.5 for WH 8102. Thus, under these conditions, MIT 9313 devotes relatively more resources to the transport of electrons away from PSII or around PSI, and less to RUBISCO, which catalyzes the limiting step in the ultimate transfer of those electrons to fix carbon. This suggests that MIT 9313 uses photosynthesis rate less for carbon fixation and more for ATP generation through cyclic electron flow around PSI than the other strains. It should also be noted from Figure 1 that this increased allocation to cytochrome b*_6_*f and PSI occurs as the cells enter their phase of most rapid growth and decreases as division slows in older cultures. Overall, the highlight ecotype of *Prochlorococcus* (MED 4) has a PSU stoichiometry more similar to the marine *Synechococcus* strain than to the low light *Prochlorococcus* strain (MIT 9313). The exception is the ratio of RUBISCO to PSII where the *Prochlorococcus* strains both have significantly (*p* < 0.0001, one way ANOVA with Tukey’s post-test) less RUBISCO per PSII than does the marine *Synechococcus*.

**Table 1:** Photophysiological Parameters.

Parameter Measured	MIT 9313	MED 4	WH 8102
σ_PSII_ (Å^2^PSII^−1^)	340 ± 17	312 ± 16	307 ± 14
ETR Capacity (e-PSII^−1^s^−1^)	155 ± 3	113 ± 12	336 ± 44
ETRmax (e-PSII^−1^s^−1^)	102 ± 2	98 ± 3	238 ± 5
E_K_ (umol photons m^−2^·s^−1^)	54 ± 3	67 ± 4	154 ± 5
μ (day^−1^)	0.25 ± 0.03	0.19 ± 0.02	0.24 ± 0.02
PSI:PSII	9 ± 5	2 ± 0.7	2 ± 0.2
Cytb*_6_*f:PSII	2.5 ± 0.5	1.3 ± 0.4	1.1 ± 0.1
Cytb*_6_*f:PSI	0.2 ± 0.2	0.7 ± 0.1	0.5 ± 0.1
RUBISCO:PSII	0.5 ± 0.1	0.8 ± 0.2	1.5 ± 0.2

The mean ± standard deviation are presented for replicates of 4–6.

### 3.3. Photosystem II Photophysiological and Electron Transport Differences among Strains

To probe possible functional consequences of the different PSU stoichiometries, PSII function and electron transport measurements were made by Fast Repetition Rate (FRR) Chlorophyll Fluorescence, summarized in Table 1. The functional absorption cross sections (σ_PSII_) for blue light for the strains are similar, ranging from 306.8 ± 13.9 to 340.5 ± 16.7 Å^2^PSII^−1^. The marine *Synechococcus*, however, shows a higher maximum PSII electron transport rate, a bigger electron transport capacity for re-oxidation of PSII after a saturating flash and a higher E_K_ than the *Prochlorococcus* strains.

**Figure 3:**
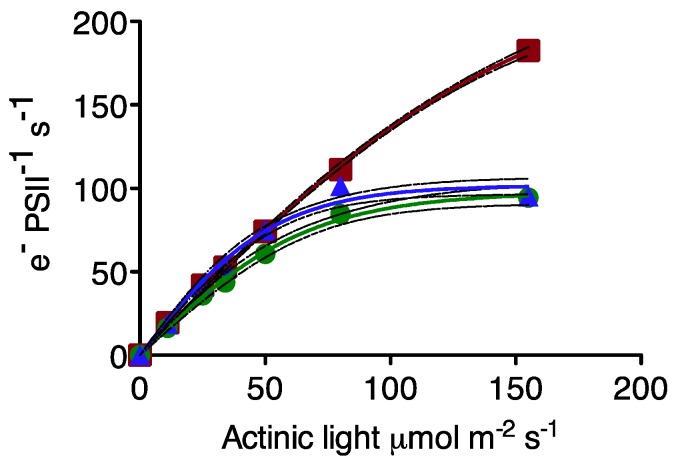
Light response of PSII electron transport (e^−^ PSII^−1^·s^−1^). *Prochlorococcus* MIT 9313: blue triangles; *Prochlorococcus* MED 4: green circles; *Synechococcus* WH8102: red squares. For clarity each plotted point represents the average of seven to eight independent determinations measured on separate cultures. The solid lines are hyperbolic tangent fits of the data; dotted lines show 95% confidence intervals on the regression.

Light response curves for PSII electron transport are shown in Figure 3. The two *Prochlorococcus* strains show very similar responses to increasing light, and show catalytic limitation of the electron transport rate by 150 μmol photons m^−2^·s^−1^. The marine *Synechococcus* strain is not catalytically limited over this irradiance range, as shown by its higher E_K_. 

**Figure 4:**
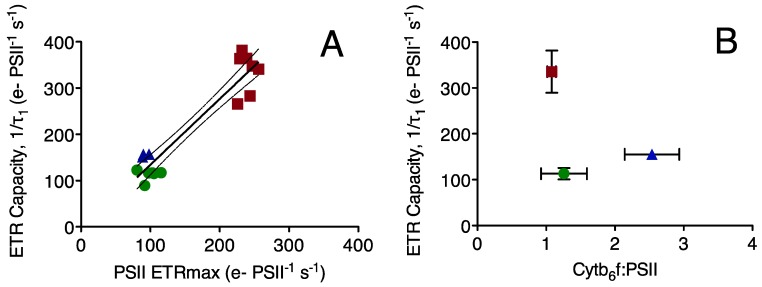

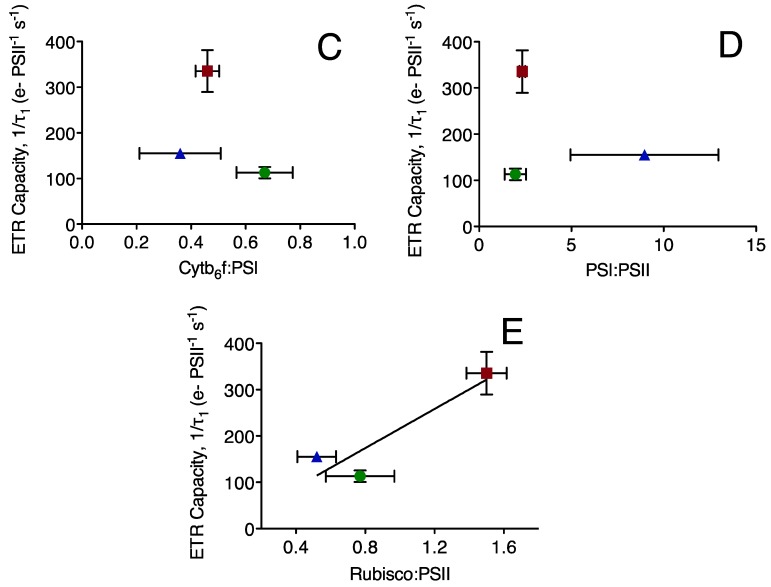
(**a**) Electron Transport Capacity away from Photosystem II 1/τ_2_ (e^−^ PSII^−1^ s^−1^) plotted *versus* maximum Electron Transport Rate for Photosystem II under saturating light (e^−^ PSII^−1^ s^−1^); each point represents a paired measurement of the two parameters on an independent culture. *Prochlorococcus* MIT 9313: blue triangles; *Prochlorococcus* MED 4: green circles; *Synechococcus* WH8102: red squares. Line shows linear regression through the pooled points from all three strains; R^2^ = 0.89, dotted lines show 95% confidence interval on the pooled regression; (**b**) Electron Transport Capacity away from Photosystem II 1/τ_2_ (e^−^ PSII^−1^ s^−1^) (n = 3–6 independent determinations, ±95% confidence interval on the y-axis) plotted *versus* the ratio of Cytb_6_f: PSII (n = 6 independent determinations, ±95% confidence interval on the x-axis);(**c**) Electron Transport Capacity away from Photosystem II 1/τ_2_ (e^−^ PSII^−1^ s^−1^) (n = 3–6 independent determinations, ±95% confidence interval on the y-axis) plotted *versus* the ratio of Cytb_6_f : PSI (n = 6 independent determinations, ±95% confidence interval on the x-axis); (**d**) Electron Transport Capacity away from Photosystem II 1/τ_2_ (e^−^ PSII^−1^ s^−1^) (n = 3–6 independent determinations, ± 95% confidence interval on the y-axis) plotted *versus* the ratio of PSI: PSII (n = 6 independent determinations, ±95% confidence interval on the x-axis); (**e**) Electron Transport Capacity away from Photosystem II 1/τ_2_ (e^−^ PSII^−1^ s^−1^) (n = 3–6 independent determinations, ± 95% confidence interval on the y-axis) plotted *versus* the ratio of RUBISCO: PSII (n = 6 independent determinations, ±95% confidence interval). Line shows linear regression; R^2^ = 0.99, dotted lines show 95% confidence interval on the pooled regression.

To determine a mechanistic basis for the differences in photosynthetic electron transport we plot the capacity for electron transport away from PSII (ETR capacity) *versus* other measured parameters (Figure 4). As expected, the ETR capacity is strongly correlated to ETRmax, the electron transport rate under saturating light, with an R^2^ of 0.89 (Figure 4A). ETR capacity is not correlated to the molar ratio of cytochrome b_6_f to PSII (Figure 4B), nor to the molar ratio of cytochrome b_6_f to PSI (Figure 4C) nor to the ratio of PSI to PSII (Figure 4D). This indicates that the capacity for electron transport away from PSII is not directly limited by the available pool of cytochrome b*_6_*f complexes serving PSII or PSI, nor by the pool of PSI extracting electrons from the inter-system electron transport chain. In fact, the strain with the lowest ratio of cytochrome b*_6_*f to PSII shows the highest rate of electron transport away from PSII. ETR capacity is, however, strongly correlated to the ratio of RUBISCO active sites (measured RbcL subunits) to PSII, with an R^2^ of 0.83 across the three strains (Figure 4E). 

### 3.4. Discussion

The photosynthetic complex immunoquantitations coupled with photophysiology presented here give new insights into the photosynthetic strategies employed by different picocyanobacterial strains grown under identical, low light conditions. The achieved growth rates for the three strains are similar, although the growth rate for MED 4 is significantly lower than for MIT 9313 and WH 8102 (*p* < 0.05), but the photophysiology and particularly the ratios of photosynthetic protein complexes are rather different. Correlations of our data indicate that the electron transport rate away from PSII is most strongly related to RUBISCO:PSII and not to relative cytochrome b*_6_*f or PSI contents. Regulation of the maximum turnover rate of the cytochrome b*_6_*f complex has been suggested as a mechanism to control the rate of transport of electrons through the plant electron transport chain [35,36]. If such a mechanism were significant in the picocyanobacteria, we would expect to see a positive correlation between the cytochrome b*_6_*f:PSII ratio and the electron transport rate away from PSII. This is not observed despite significant differences in the cytochrome b*_6_*f:PSII ratio between strains. We reported in Fraser *et al.* [37], light response curves for PSII electron transport rate in the freshwater cyanobacteria *Synechocystis* PCC 6803 and *Synechococcus* PCC 7942. We showed that despite having identical cytochrome b*_6_*f:PSII ratios under iron replete conditions, the PSII electron transport rates were much higher in *Synechocystis* than *Synechococcus* indicating that similar ratios of these components of the electron transport chain do not correlate to electron transport rates in the freshwater cyanobacteria. Furthermore, upon iron starvation, while the ratio of cytochrome b*_6_*f:PSII dropped significantly in *Synechocystis*, the electron transport rate did not, and in *Synechococcus* the electron transport rate decreased but the ratio of cytochrome b*_6_*f:PSII did not. Clearly, the content of cytochrome b*_6_*f relative to PSII does not predict transport of electrons away from PSII in neither freshwater nor marine cyanobacteria. Rather, our data suggest that the sustained rate of electron transport away from PSII is correlated with the capacity of the cells to perform the rate-limiting step of the Calvin cycle, the ultimate electron sink.

Sukenik *et al*. [38] demonstrated that for the eukaryotic alga *Dunaliella tertiolecta*, the light-saturated photosynthetic rate was limited by the molar ratio of RUBISCO to the photosynthetic unit (PSU). In that study the authors present the results for *Dunaliella* grown under a range of growth irradiances from 80 to 1900 μmol photons m^−2^·s^−1^. In that strain, the molar ratios of the components of the photosynthetic unit (PSII, plastoquinone (PQ), cytochrome b*_6_*f and PSI) remained constant while the number of photosynthetic units per cell decreased with increased growth irradiance. Furthermore, the authors report constant levels of RUBISCO protein and activity resulting in an increase in the RUBISCO to PSU ratio. The authors report a strong linear relationship between 1/τ (ETRmax) and the RUBISCO to PSU ratio and suggest that the RUBISCO pool relative to the number of PSU limits the overall rate of photosynthesis in this organism [38]. Furthermore, we have recently shown that for a series of six eukaryotic marine phytoplankters grown under a range of irradiances, photoacclimation was achieved through an increase in the RUBISCO to photosystem ratio [39]. It is important to note that in that study five species achieved this change in ratio through a decrease in the number of photosystems, while one diatom (*Skeletonema marinoi*) increased the RUBISCO content. It is interesting to compare these results to those presented here for picocyanobacteria where we report strikingly different PSU compositions (molar ratios) between the strains examined, but still the RUBISCO to PSII ratio appears to limit the electron transport rate. Our data allow us to correlate the electron transport rate to several ratios of components of the PSU, indicating that it is the ratio of RUBISCO to PSII that best relates to electron transport rate. It would be interesting to determine whether the picocyanobacterial strains examined here would vary similarly with differing growth irradiances.

Kulk *et al.* [40] reported the effects of growth temperature on the photophysiology of the two *Prochlorococcus* strains analyzed here, showing that changes in electron transport cannot fully explain differences in growth rate. It should be noted that the authors caution that their calculations of absolute electron transport rates assume a PSI:PSII ratio of 1:1 and that to the extent PSI:PSII exceeds 1:1, their estimates of electron transport may be over estimates. Our results suggest this is likely true, as we measure PSI:PSII ratios of 9:1 for MIT 9313 and 2:1 for MED 4. Kulk *et al*. [40] also report spectrally weighted mean specific absorption coefficients (ā* m^2^ mg^−1^ chl *a*) for the two *Prochlorococcus* strains grown under conditions similar to those reported here. They report values of 0.033 m^2^ mg^−1^chl *a* for MIT 9313 and 0.013 m^2^ mg^−1^ chl *a* for MED 4 with MIT 9313 having a higher blue:red absorbance ratio. When taken together with our data, we speculate that the increased specific absorption coefficient per chlorophyll for MIT 9313 represents increased absorption by PSI in this strain, likely resulting in cyclic electron flow around PSI. Indeed, if this is the case, more than half of the photons absorbed in MIT 9313 are absorbed by PSI.

High cellular contents of PSI and cytochrome b*_6_*f come at a considerable nutrient cost in the form of iron requirements. Using the iron stoichiometries for the complexes reported in their crystal structures (3 Fe per PSII monomer [41]; 6 Fe per cytochrome b*6*f monomer [42]; and 12 Fe per PSI monomer [43]) the iron content of the PSU (normalized to PSII) for MIT 9313 is 125.7 Fe atoms, whereas it is 34.1 Fe atoms for MED 4 and 37.6 Fe atoms for WH 8102. We thus speculate that the growth rate afforded by MIT 9313 under nutrient (including iron) replete conditions, perhaps utilizing cyclic electron flow around PSI for extra ATP generation, would be lost under iron limiting conditions. The data presented here provide no direct evidence for cyclic electron flow as we were unable to measure this for the current work, however given the considerations reviewed in Behrenfeld and Milligan [44] the extreme PSI:PSII ratio measured in MIT 9313 suggests that linear electron transport would be out of balance in this strain. Measurement of the content and activity of other terminal oxidases in these strains could shed light on this in greater detail. A number of *Prochlorococcus* isolates have been reported to locate to low iron regions of the oceans [45] and it would be interesting to probe the photosynthetic apparatuses of these isolates similarly. 

## 4. Conclusions 

The cellular pool of the RUBISCO enzyme, catalyzing the rate-limiting step of carbon fixation, appears to limit electron transport away from PSII, rather than the intermediary electron transport complexes bound to the thylakoid membrane in these picocyanobacteria. This is reflected in the ETR light response curves in Figure 3 which show catalytic limitation of ETRmax under moderate light, in the *Prochlorococcus* strains with the low RUBISCO:PSII ratios. 

Furthermore, high PSI:PSII ratios in MIT 9313 coupled to low contents of RUBISCO suggest that cyclic electron flow around PSI may contribute to higher growth rates in this strain. This growth comes at a cost if iron requirements are factored in, as the PSU of MIT 9313 requires more than three times as much iron as those of MED 4 and WH 8102.

## Supplementary Materials

Supplementary materials can be accessed at: http://www.mdpi.com/2075-1729/5/1/403/s1.
