# Supplementary material for: The RUBISCO to Photosystem II Ratio Limits the Maximum Photosynthetic Rate in Picocyanobacteria
Source: Life (Basel). 2015 Feb 4;5(1):403–17. doi: 10.3390/life5010403 (PMC4390859; doi:10.3390/life5010403)
Supplement: Supplementary file 1 [file life-05-00403-s001.pdf]

Supplementary Material

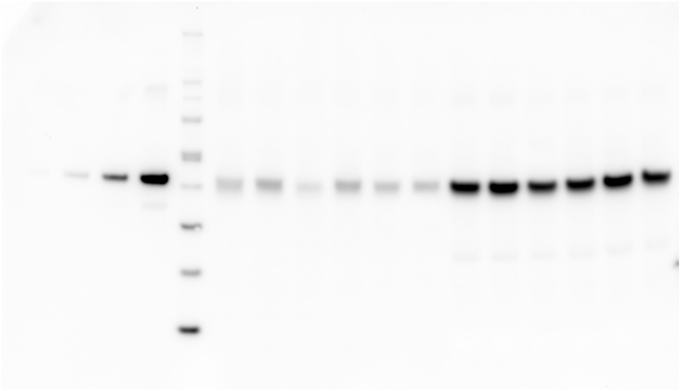

(A)

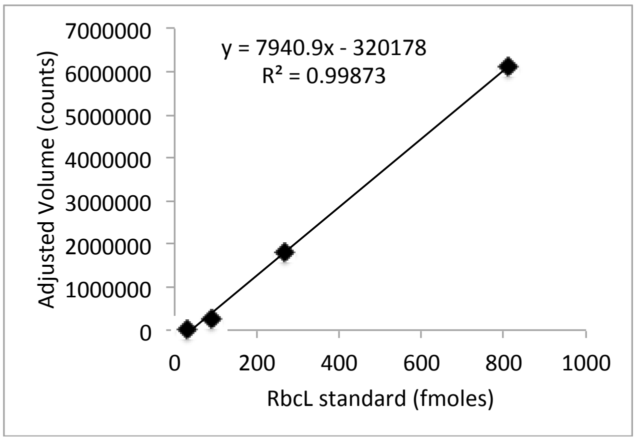

(B)

Sample	Volume (counts)	fmoles RbcL in load	Ug protein loaded	fmoles RbcL/ug total protein
MED4 A	1110122	180.3	3	60.1
MED4 B	1479660	226.7	3	75.6
MED4 C	515060	105.4	3	35.1
MED4 D	1378832	214.1	3	71.4
MED4 E	958984	161.3	3	53.8
MED4 F	1006044	167.2	3	55.7
WH 8102 A	5311800	708.7	10	70.9
WH 8102 B	6120946	810.5	10	81.0
WH 8102 C	4535596	611.1	10	61.1
WH 8102 D	5054452	676.3	10	67.6
WH 8102 E	6070064	804.1	10	80.4
WH 8102 F	4805398	645.0	10	64.5

(C)

Figure S1. The protein immunoquantitation method is illustrated in this figure. (**A**) a representative blot (Lane 1: 30 fmoles RbcL; Lane 2: 90 fmoles RbcL; Lane 3: 270 fmoles RbcL; Lane 4: 810 fmoles RbcL; Lane 5: Molecular Weight Marker; Lanes 6–11: 3 ug loads (total cellular protein) of *Prochlorococcus marinus* sp. MED 4; Lanes 12–17: 10 ug loads (total cellular protein) of *Synechococcus* WH 8102 detected with anti-RbcL antibodies as described in the Materials & Methods section); (**B**) Standard curve of Adjusted Volume versus fmoles of RbcL loaded; (**C**) Table of values calculated from the standard curve.

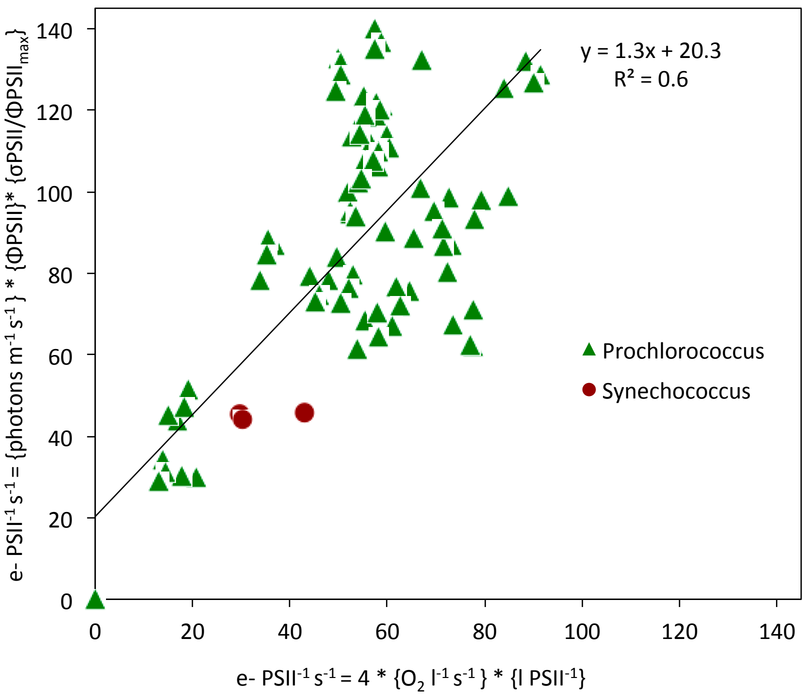

Figure S2. A correlation of the fluorescence based estimate of electron transport per PSII per s (Y axis) versus an oxygen-evolution based estimate of electron transport per PSII per s (X axis), for *Prochlorococcus* MIT 9313 and MED 4 samples (green triangles) and *Synechococcus* WH 8102 samples (red circles).

As expected the chlorophyll fluorescence estimate of PSII electron transport is well correlated with the oxygen based measure. For routine experiments we use the much faster and more sensitive fluorescence based estimate. Furthermore, our independent estimates of the rate of electron transport flow away from PSII, based upon fluorescence decay after a saturating flash (Materials & Methods; Figure 4a), also correlate closely with our fluorescence based and oxygen based estimates of maximum electron transport.

Cell culture samples were loaded into a 1 cm spectrophotometer cuvette and placed in the SuperHead optical unit of a PSI 3500 chlorophyll fluorometer (PSI, Czech Republic). Each culture sample was exposed to a range of actinic light between 0 and 260 µmol photons m^{-2} s^{-1}. After 5 min exposure to a light level we applied a Fast Repetition Rate fluorescence single turnover induction train of 40 flashlets of 1.2 µs duration separated by 2 µs dark, as described in the Materials and Methods. We then followed [33] to estimate Photosystem II electron transport across the range of light levels as:

$$e^{-}\ PSII^{-1}\ s^{-1} = \sigma_{PSII}'/(F_V/F_M) \times \Phi PSII \times I\ (\text{photons } m^{-2}\ s^{-1})$$

We simultaneously measured the instantaneous concentration of dissolved oxygen in the culture sample using a FireSting Optode (PyroScience, Germany) solid state oxygen monitor. We used the dark respiration and the net oxygen evolution in the light to estimate O_2 l^{-1} s^{-1}, and multiplied by 4 e^{-}/O_2 to express e^{-} l^{-1} s^{-1} under each applied actinic light level. Prior to, and subsequent to, the exposure to the series of actinic light levels, we estimated the concentration of active PSII l^{-1} using a flash yield protocol following [33]. Briefly, we applied a low level of actinic light to induce electron transport. We then interrupted the actinic light and applied a train of saturating single turnover flashes

of 25 μs duration separated by 25 ms dark, sufficient for downstream electron transport to carry the photochemically generated electron away from PSII, leaving each PSII centre open and ready to accept the next saturating flash. Since each active PSII can evolve 1 O_2 for every 4 flash cycles, we can extract the concentration of active PSII from the resulting gross slope of oxygen evolution. We routinely test that the single turnover flash train is delivering sufficient excitation to saturate the oxygen evolution response, to ensure that each active PSII is indeed excited upon each flash, and that the spacing of flashes is sufficient to allow removal of electrons (~1 ms timescale) but close enough to preempt decay of the S-state progression (~1 s timescale). We can then multiply e^{-} l^{-1} s^{-1} by l $PSII^{-1}$ to derive an independent estimate of e^{-} $PSII^{-1}$ s^{-1}.
